# HDR Endorectal/Endoluminal Brachytherapy Boost in Rectal Organ Preservation: A Systematic Review and Meta-Analysis

**DOI:** 10.3390/cancers18091494

**Published:** 2026-05-06

**Authors:** Yuanjie Cao, Chen Li, Baozhong Zhang, Jie Chen

**Affiliations:** 1 Department of Radiation Oncology, Tianjin Medical University Cancer Institute & Hospital, Tianjin 300060, China; cyjro325@gmail.com (Y.C.); lichen.radonco@tmu.edu.cn (C.L.); 2 Tianjin’s Clinical Research Center for Cancer, Key Laboratory of Cancer Prevention and Therapy, National Clinical Research Center for Cancer, Tianjin 300060, China

**Keywords:** rectal cancer, organ preservation, non-operative management, HDR brachytherapy, endorectal brachytherapy, dose escalation

## Abstract

Background and purpose: Organ-preservation strategies are increasingly being incorporated into rectal cancer management, but the role of HDR endorectal/endoluminal brachytherapy boost remains less well defined than that of broader non-operative treatment pathways. Existing literature is frequently mixed with contact X-ray brachytherapy series, neoadjuvant protocols with planned surgery, or heterogeneous watch-and-wait cohorts, limiting the interpretation of this specific strategy. Materials and methods: We performed a PROSPERO-registered systematic review and meta-analysis of studies evaluating definitive-intent external beam radiotherapy (EBRT), with or without chemotherapy, followed by HDR endorectal/endoluminal brachytherapy boost in histologically confirmed rectal adenocarcinoma managed without planned surgery. Pooled analyses were performed for clinical complete response (cCR) and late grade ≥3 gastrointestinal (GI) toxicity. Regrowth/local failure outcomes were synthesized descriptively because of heterogeneity in endpoint definitions, denominator selection, and follow-up structure. Results: Six studies were included in the quantitative evidence base, with one additional small feasibility report summarized narratively. The pooled cCR proportion was 69.2% (95% confidence interval [CI], 43.7–86.6). The pooled proportion of late grade ≥3 GI toxicity was 18.1% (95% CI, 10.9–28.6). Reported regrowth/local failure outcomes were not suitable for formal pooling because of inconsistent definitions, differing denominator structures, and non-uniform follow-up frameworks across studies. Conclusion: Current evidence suggests that EBRT plus HDR endorectal/endoluminal brachytherapy boost may represent a selective organ-preservation strategy for carefully chosen patients with rectal adenocarcinoma, particularly where surgery is not feasible or not desired. Its broader clinical use remains limited not only by the small size of the evidence base, but also by fragmented endpoint definitions, inconsistent denominator reporting, and insufficiently standardized durable local-control outcomes. These findings support cautious interpretation of the current evidence and highlight priorities for future prospective studies in rectal cancer management.

## Simple Summary

Rectal cancer treatment is increasingly moving toward approaches that can control the tumor while preserving the rectum and avoiding major surgery in carefully selected patients. One possible strategy is to combine external radiation treatment with an internal radiation boost delivered close to the tumor through an endorectal or endoluminal brachytherapy applicator. However, the evidence for this specific approach has been scattered across small studies with different patient groups, treatment schedules, and outcome definitions. In this systematic review and meta-analysis, we summarized the available clinical evidence on this strategy for rectal adenocarcinoma managed without planned surgery. The findings suggest that this approach may produce meaningful complete response rates in selected patients, but severe late bowel toxicity and inconsistent reporting remain important concerns. Future studies should use clearer patient selection, standardized outcome definitions, and longer follow-up.

## 1. Introduction

Organ preservation has become an increasingly important therapeutic objective in rectal cancer. For selected patients, avoiding total mesorectal excision may reduce the risks of permanent stoma, bowel dysfunction, and treatment-related impairment in quality of life [1,2,3]. At the same time, local treatment intensification strategies are increasingly being considered within multidisciplinary rectal cancer care, particularly when balancing oncologic control, treatment-related morbidity, and patient preference. Non-operative management is therefore not a single strategy but a spectrum of pathways that differ substantially in intent, patient selection, local-treatment intensification, and tolerance for uncertainty regarding durable tumor control. Within this evolving landscape, the challenge is no longer simply whether surgery can be avoided in some patients, but how local control, toxicity, salvageability, and functional outcomes should be balanced across different organ-preservation approaches [4,5]. Recent prospective organ-preservation studies, including OPRA and OPERA, have reinforced both the clinical appeal and the complexity of non-operative pathways in rectal cancer, underscoring the need to define how different local-intensification strategies may fit within this evolving treatment landscape [6,7]. For clarity, the present review focuses on histologically confirmed rectal adenocarcinoma, which constitutes the disease entity addressed by contemporary rectal organ-preservation studies; anal squamous cell carcinoma, anal canal cancer, and other non-adenocarcinoma anorectal malignancies were outside the scope of this analysis.

Among local intensification strategies, HDR endorectal/endoluminal brachytherapy boost occupies a distinct but incompletely defined position. Unlike contact X-ray brachytherapy, HDR endorectal/endoluminal brachytherapy is delivered through a different technical platform, with different dose-distribution characteristics and a different evidence base [8,9]. In principle, it offers the possibility of tumor dose escalation while limiting exposure of surrounding normal tissues, and has been explored in medically inoperable patients, patients declining surgery, and selected organ-preservation cohorts treated without planned resection [9,10,11,12]. Yet, these clinical scenarios are not interchangeable, and the relevance of this strategy may differ substantially across them [4,8,13].

Interpretation of the existing literature is complicated by several factors. First, available studies are few and mostly small. Second, treatment regimens vary across EBRT dose-fractionation, chemotherapy use, and brachytherapy schedules. Third, the literature is often embedded within broader non-operative management discussions that combine conceptually distinct pathways [3,14,15]. Finally, reported outcomes are unevenly structured: early clinical response is more often described than durable local control, and denominator definitions for response, toxicity, and regrowth/local failure frequently differ across reports. As a result, the apparent promise of this strategy has remained difficult to position within contemporary rectal cancer management.

We therefore performed a systematic review and meta-analysis focused specifically on EBRT plus HDR endorectal/endoluminal brachytherapy boost used with definitive non-operative intent in rectal adenocarcinoma. Our aims were not only to synthesize cCR and severe late GI toxicity, but also to clarify the current clinical position of this strategy within modern organ-preservation pathways and to identify the methodological barriers that currently limit stronger inference regarding durable local control.

## 2. Materials and Methods

### 2.1. Protocol and Reporting

This systematic review and meta-analysis were conducted in accordance with PRISMA 2020 [16]. The review protocol was registered in PROSPERO, the International Prospective Register of Systematic Reviews (CRD420261339587).

### 2.2. Eligibility Criteria

Studies were eligible if they included patients with histologically confirmed rectal adenocarcinoma treated with definitive-intent EBRT, with or without chemotherapy, followed by HDR endorectal/endoluminal brachytherapy boost, and managed without planned surgery. Both medically inoperable or frail patients and surgery-refusing or intentional organ-preservation cohorts were eligible, provided that the overall treatment pathway was non-operative in intent. We excluded studies primarily addressing anal squamous cell carcinoma, anal canal cancer, non-adenocarcinoma histologies, neoadjuvant or preoperative brachytherapy series with planned resection, contact X-ray brachytherapy-only studies, reports without a combined EBRT plus HDR endorectal/endoluminal boost approach, duplicate datasets, and reports from which relevant outcomes could not be extracted.

### 2.3. Search Strategy and Study Selection

PubMed, Embase, and CENTRAL were searched for studies published between 2010 and 2026 using terms related to rectal cancer, external beam radiotherapy, HDR brachytherapy, endorectal brachytherapy, endoluminal brachytherapy, and non-operative management, including organ-preservation strategies. The 2010 lower boundary was chosen to focus on contemporary HDR endorectal/endoluminal brachytherapy practice, modern imaging-based rectal cancer assessment, and organ-preservation frameworks relevant to current multidisciplinary management; earlier reports were expected to differ substantially in technical platforms, imaging assessment, dose reporting, and endpoint definitions. Two reviewers independently screened titles and abstracts, followed by full-text assessment of potentially eligible records. Disagreements were resolved by discussion and, when necessary, adjudication by a third reviewer. Data extraction was performed using a standardized form and cross-checked for accuracy. Reference lists of relevant reviews and included studies were also screened to identify additional eligible reports. No language restrictions were applied during the initial search; however, only studies with sufficient extractable data for analysis were included in the final synthesis.

### 2.4. Data Extraction and Endpoints

For each included study, we extracted study design, cohort context, EBRT and chemotherapy details, HDR endorectal/endoluminal boost parameters, follow-up, and outcome data. The primary endpoints for quantitative synthesis were cCR and late grade ≥3 GI toxicity. Regrowth/local failure, follow-up duration, and denominator type were extracted descriptively. Because outcome definitions were not uniform across studies, particular attention was paid to whether numerators and denominators referred to all treated patients, evaluable subsets, or surveillance-entry populations.

### 2.5. Risk-of-Bias Assessment

Risk of bias was assessed according to the study design. Case series and non-comparative cohorts were evaluated using the JBI critical appraisal checklist for case series [17]. The randomized trial arm was assessed using RoB 2 [18]. Item-level evidence and summary judgements for the JBI case-series assessment are provided in the Supplementary Material, with the visual summary shown in Supplementary Figure S1; RoB 2 is presented as a narrative summary because only a single trial arm was eligible for that assessment. Methodological quality and risk-of-bias assessments were performed independently by two reviewers, with disagreements resolved by discussion and, when necessary, adjudication by a third reviewer.

### 2.6. Statistical Analysis

Proportions for cCR and late grade ≥3 GI toxicity were pooled using random-effects models on the logit scale with DerSimonian-Laird τ^2^ estimation [19]. Heterogeneity was quantified by I^2^ [20]. Regrowth/local failure was summarized descriptively because of inconsistent endpoint definitions, denominator structures, and follow-up reporting. Additional robustness analyses were prespecified for the pooled cCR and late grade ≥3 GI toxicity endpoints. Leave-one-out analyses were performed to assess the influence of individual studies on the pooled estimates. Predefined sensitivity analyses restricted to studies with the most consistent endpoint definitions and denominator reporting were undertaken for both cCR and toxicity. For cCR, a prediction interval was estimated to reflect the expected variability across future comparable cohorts. For late grade ≥3 GI toxicity, an alternative sparse-data model was explored as a sensitivity analysis. Meta-analyses were conducted using a random-effects model on the logit scale. Statistical analyses were performed in R (version 4.5.1, R Foundation for Statistical Computing, Vienna, Austria), using standard meta-analytic packages.

## 3. Results

### 3.1. Study Selection

**Figure 1:**
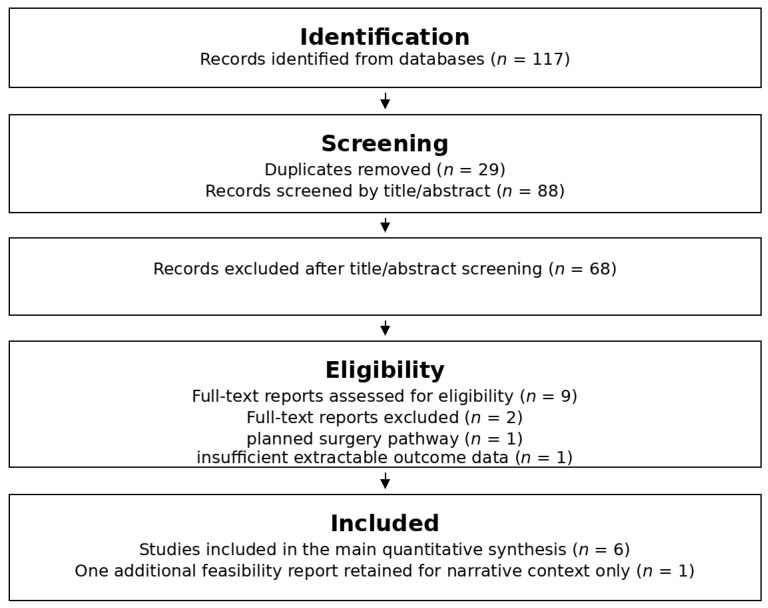
PRISMA 2020 flow diagram of study identification, screening, and inclusion.

The database search identified 117 records, including 29 from PubMed, 58 from Embase, and 30 from CENTRAL. After removal of 29 duplicates, 88 unique records underwent title/abstract screening. Nine full-text reports were assessed for eligibility. Six studies met the criteria for the overall quantitative synthesis [10,11,12,21,22,23], and one additional small feasibility report was retained for narrative context only [24] (Figure 1). Endpoint-specific pooled analyses used available studies, with cCR pooled from five studies and late grade ≥3 GI toxicity pooled from five studies.

### 3.2. Overview of the Included Evidence

The included studies consisted predominantly of small institutional cohorts, together with one randomized trial arm and one registry-based series. Across studies, the clinical context was heterogeneous, spanning medically inoperable or frail patients, patients refusing surgery, and organ-preservation cohorts treated without planned resection. Treatment delivery also varied with respect to EBRT dose-fractionation, chemotherapy use, and HDR endorectal/endoluminal brachytherapy boost schedules. Detailed study- and treatment-level characteristics are provided in Table 1 and Table 2.

### 3.3. Pooled Efficacy and Toxicity Outcomes

Because the included studies used different analysis populations, all pooled and descriptive outcomes were interpreted according to the denominator reported in each source study. For cCR, denominators included treated/ITT populations in some studies and response-evaluable populations in others. For late grade ≥3 GI toxicity, denominators included treated/ITT, toxicity-evaluable, or responder-only populations. These denominator types are explicitly reported in Table 3 and were considered when determining whether formal pooling was appropriate.

**Figure 2:**
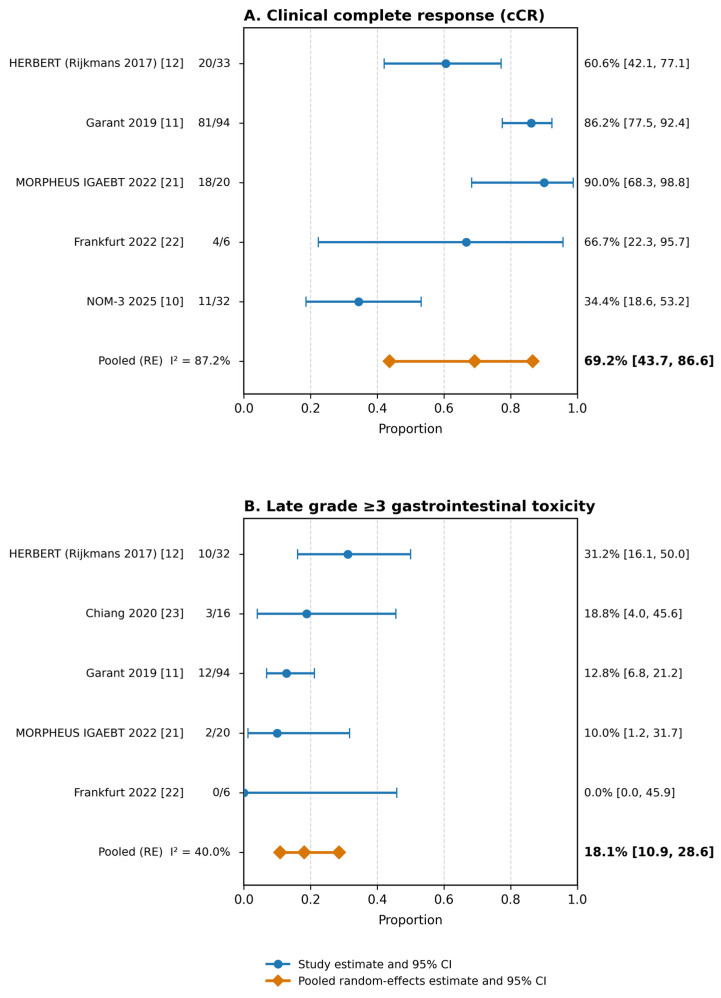
Pooled proportions of (**A**) clinical complete response (cCR) and (**B**) late grade ≥ 3 gastrointestinal toxicity after EBRT (±chemotherapy) plus HDR endorectal/endoluminal brachytherapy boost. Overall quantitative synthesis included six studies; endpoint-specific pooled analyses used all studies with extractable data (cCR *n* = 5; late grade ≥ 3 GI toxicity *n* = 5). Blue circles and horizontal lines indicate study-level estimates and 95% confidence intervals. Orange diamonds and horizontal lines indicate pooled random-effects estimates and 95% confidence intervals. Bold values indicate pooled random-effects estimates. Study-level denominators were used as reported in the original studies and are detailed in Table 3.

Using the five studies with extractable cCR data for quantitative synthesis, the pooled proportion of clinical complete response (cCR) was 69.2% (95% CI 43.7–86.6%), with substantial between-study heterogeneity (I^2^ = 87.2%; Figure 2A).

Late severe gastrointestinal (GI) toxicity was reported less consistently than response. Using the five studies with extractable late grade ≥3 GI toxicity data, the pooled proportion of late grade ≥3 GI toxicity was 18.1% (95% CI 10.9–28.6%), with moderate heterogeneity (I^2^ = 40.0%; Figure 2B).

Taken together, these pooled findings indicate that EBRT followed by HDR endorectal/endoluminal brachytherapy boost was associated with a pooled cCR proportion of 69.2% in selected non-operative rectal cancer cohorts, while late severe GI toxicity remained clinically relevant. These estimates should be interpreted as summaries of extractable published data rather than as estimates derived from fully harmonized ITT populations.

### 3.4. Descriptive Findings for Regrowth/Local Failure

**Figure 3:**
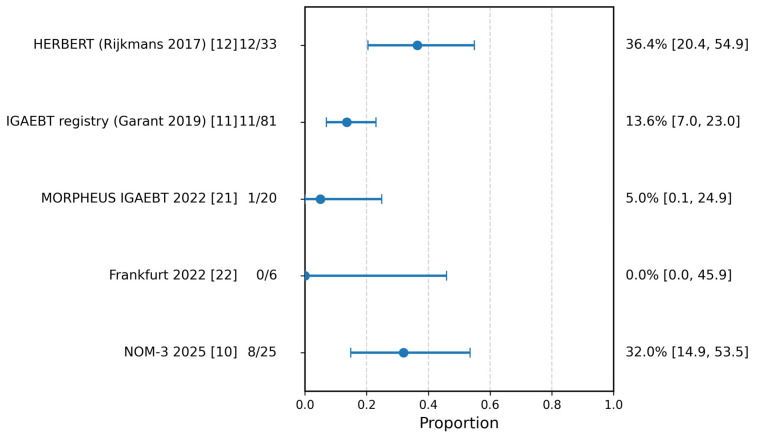
Regrowth/local failure proportions among reported denominators, shown descriptively as reported by individual studies. Because denominator definitions differed across studies, no pooled estimate was calculated. Study-specific denominator information is detailed in Table 3.

Reported regrowth/local failure proportions varied across studies and denominator definitions, ranging from 0/6 in the Frankfurt series to 11/81 in the Garant registry, 12/33 in the HERBERT cohort, 1/20 in MORPHEUS, and 8/25 in NOM-3; the latter was back-calculated from the reported percentage and denominator because absolute counts were not directly stated in the original report (Figure 3; Table 3).

These proportions should be interpreted cautiously because denominators were not comparable across studies. In particular, some reports described local events among all treated patients, whereas others reported regrowth only among patients entering surveillance after cCR or near-cCR. Accordingly, the descriptive range reflects both clinical variability and reporting heterogeneity rather than a directly comparable estimate of local control failure.

### 3.5. Follow-Up and Evidence Limitations Relevant to Outcome Interpretation

Follow-up varied across cohorts and was generally limited for robust assessment of long-term durability. This is particularly relevant for regrowth/local failure, for which event ascertainment depends on both surveillance intensity and duration of observation.

Several evidence limitations are important when interpreting the present results. First, most cohorts were small. Second, patient populations and treatment intent were heterogeneous. Third, denominator definitions differed across outcomes, including response-evaluable subsets, treated cohorts, and surveillance-entry populations. Finally, local regrowth/local failure endpoints were not reported in a sufficiently standardized manner to support formal meta-analysis. These limitations do not negate the pooled estimates for cCR and late grade ≥ 3 GI toxicity, but they may reduce precision, limit generalizability, and constrain inference regarding the durability of local tumor control.

### 3.6. Sensitivity and Robustness Analyses

Each robustness analysis used endpoint-specific available study sets as defined in Supplementary Table S1. For cCR, sequential omission analyses yielded pooled estimates ranging from 63.0% to 78.2%, supporting the overall response signal despite the expected variability of a small and heterogeneous evidence base (Supplementary Figure S2). The corresponding 95% confidence intervals ranged from 36.7–83.3% to 58.7–90.1%, and the prediction interval indicated substantial expected variability across future comparable cohorts (Supplementary Table S2).

For late grade ≥3 GI toxicity, leave-one-out analyses suggested that no single study fully accounted for the pooled estimate, with sequential omission analyses yielding pooled estimates from 13.0% to 20.5% (Supplementary Figure S3). Specifically, omission of HERBERT (Rijkmans 2017) [12], Garant 2019 [11], Chiang 2020 [23], MORPHEUS IGAEBT 2022, and Frankfurt 2022 yielded pooled estimates of 13.0%, 20.5%, 16.6%, 18.6%, and 17.8%, respectively. The corresponding 95% confidence intervals are reported in Supplementary Table S3. An alternative sparse-data model yielded a directionally similar estimate of 17.3% (95% model-based interval 10.0–26.0; Supplementary Table S4).

### 3.7. Ongoing Prospective Studies

A complementary search of ClinicalTrials.gov, queried on 6 March 2026, identified a small number of registered prospective studies relevant to endorectal/endoluminal HDR brachytherapy strategies in rectal cancer. These studies are summarized in Supplementary Table S5.

Overall, the registered studies indicate continuing prospective and technical development in this field, but they do not materially alter the interpretation of the current pooled evidence, which remains driven by small published cohorts with heterogeneous reporting structures.

## 4. Discussion

This systematic review and meta-analysis suggests that EBRT plus HDR endorectal/endoluminal brachytherapy boost shows a substantial response signal in selected non-operative rectal cancer cohorts, with a pooled cCR proportion of 69.2%, but also a clinically relevant pooled proportion of late grade ≥3 GI toxicity of 18.1%. These findings are important not because they establish this approach as a standard pathway, but because they help define where this strategy may currently fit within the broader organ-preservation management of rectal cancer. Within the broader organ-preservation landscape shaped by studies such as OPRA and OPERA, the current question is not simply whether local treatment intensification can improve response, but whether it can do so with acceptable toxicity and durable local control in clearly defined patient subsets [6,7].

The central message of the available evidence is not simply that response can occur, but that the present literature remains structurally insufficient for confident conclusions about durable local control. Regrowth/local failure reporting was too inconsistent in endpoint definition, denominator structure, and surveillance framework to support formal pooling [5,25]. This limitation is clinically important rather than merely technical. In organ-preservation pathways, early cCR is only an intermediate milestone; the more clinically meaningful question is whether local tumor control can be maintained under surveillance without unacceptable toxicity or compromised salvage options [26]. The current evidence base does not yet answer that question with sufficient consistency.

A further reason why this literature requires careful interpretation is that the included cohorts represent distinct clinical scenarios rather than a single patient population. Medically inoperable or frail patients, patients refusing surgery, and intentional organ-preservation cohorts in potentially operable disease differ in baseline treatment goals, acceptable trade-offs, competing risks, and thresholds for uncertainty. Accordingly, the clinical relevance of EBRT plus HDR endorectal/endoluminal brachytherapy boost should not be assumed to be uniform across these groups. The present data support a selective role for this strategy, but not an indiscriminate one.

Toxicity remains a major consideration when interpreting the role of this approach. Although response outcomes were encouraging, the pooled proportion of late grade ≥3 GI toxicity was clinically relevant. This is particularly important in a population for whom treatment may be selected specifically to avoid the morbidity of surgery, permanent stoma, or major functional compromise [3,5]. In this context, severe late bowel toxicity is not merely a treatment complication; it directly undermines one of the principal reasons for choosing a non-operative strategy. A non-operative pathway is unlikely to remain attractive if local escalation is accompanied by substantial severe late toxicity.

Durability of local control remains less certain than initial response. Regrowth/local failure was reported across several studies, but definitions, denominators, and follow-up windows varied sufficiently to preclude formal pooling. This limitation is clinically important. In organ-preservation pathways, early cCR is not the sole endpoint of interest; sustained local control under surveillance is arguably the more meaningful outcome [4,13]. A pooled estimate would have implied a level of comparability that the underlying studies do not support. Therefore, the present evidence supports cautious descriptive interpretation rather than a potentially misleading pooled summary of local failure risk.

**Figure 4:**
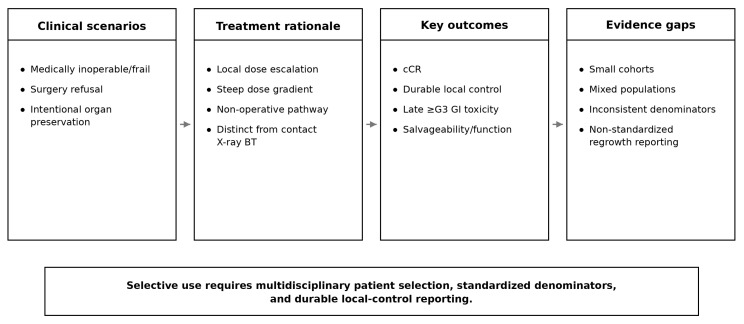
Clinical positioning and evidence gaps of EBRT plus HDR endorectal/endoluminal brachytherapy in rectal organ preservation. Schematic summary of the clinical scenarios, treatment rationale, key outcomes, and evidence gaps relevant to EBRT plus HDR endorectal/endoluminal brachytherapy as a selective organ-preserving strategy. The figure emphasizes that this approach has been explored across clinically distinct populations and should be interpreted in light of small cohorts, mixed patient groups, inconsistent denominator definitions, and non-standardized regrowth/local-failure reporting. Abbreviations: BT, brachytherapy; cCR, clinical complete response; EBRT, external beam radiotherapy; GI, gastrointestinal; HDR, high-dose-rate.

These considerations underscore the importance of patient selection. The studies included in this review encompassed distinct clinical scenarios, including medically inoperable or frail patients, patients refusing surgery, and intentional organ-preservation cohorts in potentially operable disease. These groups are not interchangeable. In frail or inoperable patients, the threshold for accepting uncertainty in local durability may differ from that in younger or surgically eligible patients pursuing organ preservation by choice. Similarly, treatment tolerance, competing risks, and salvage options vary across these populations. The present evidence therefore supports the view that EBRT plus HDR endorectal/endoluminal brachytherapy boost should be considered a selective rather than generalizable organ-preservation strategy. This interpretation is consistent with broader guideline-based principles favoring individualized, multidisciplinary selection in rectal cancer organ preservation [1,2]. Multidisciplinary evaluation, careful counseling, and structured surveillance are likely to be essential prerequisites for its use [4,13]. Future prospective studies should avoid collapsing these scenarios into a single organ-preservation cohort without prespecified population stratification. This clinical positioning framework is summarized in Figure 4, which highlights why patient selection, outcome priorities, and evidence interpretation should not be collapsed into a single non-operative category.

These considerations also depend on an appropriate diagnostic work-up before any organ-preserving strategy is considered. Histological confirmation, pelvic MRI, endoscopic assessment, and systemic staging are required to distinguish rectal adenocarcinoma from other anorectal malignancies and to define tumor site, stage, operability, and suitability for local or non-operative treatment approaches [1,2].

It is also important to emphasize that a non-operative strategy should not be interpreted as a substitute for optimal surgery in patients who are fit for resection. In high-volume tertiary centers, modern rectal cancer surgery, including robotic total mesorectal excision, nerve-sparing techniques, sphincter-preserving approaches, and intraoperative fluorescence-guided assessment with indocyanine green, has improved the feasibility of safe, well-perfused anastomoses and functional preservation [1,2,27]. Therefore, EBRT plus HDR endorectal/endoluminal brachytherapy boost should be positioned as a selective strategy for patients in whom standard surgery is declined, contraindicated, or associated with substantial functional or perioperative risk, rather than as a general replacement for contemporary surgical management.

Transanal local excision, including transanal endoscopic microsurgery or transanal minimally invasive surgery, may represent an organ-preserving option for carefully selected early-stage lesions or selected residual disease after neoadjuvant treatment [28]. However, this approach was outside the scope of the present review, which specifically evaluated definitive-intent EBRT plus HDR endorectal/endoluminal brachytherapy boost in non-operative pathways. Future organ-preservation studies should more clearly distinguish non-operative radiotherapy-based intensification from local surgical excision strategies, because these approaches differ in patient selection, oncological assumptions, morbidity profile, and salvage implications.

The heterogeneity observed in this review is also informative. Differences in EBRT dose-fractionation, use of chemotherapy, boost schedules, response assessment, and denominator definitions likely contributed to variation in pooled estimates. In particular, studies differed in whether outcomes were reported for all treated patients, evaluable subsets, or patients entering non-operative surveillance. This problem is especially relevant for regrowth/local failure, where denominator choice materially alters interpretation. More broadly, reporting heterogeneity may be as influential as clinical heterogeneity in shaping the apparent between-study differences. Such inconsistencies are common in emerging organ-preservation literature, but they limit cross-study comparability and reduce confidence in pooled inference [5]. In this setting, the principal barrier to stronger clinical interpretation is not simply the small size of the evidence base, but the lack of standardized endpoint definitions, denominator selection, and durable local-control reporting across studies.

Additional robustness analyses strengthen the interpretation of the pooled findings while also clarifying their limits. For cCR, leave-one-out analyses supported the overall response signal, but the wide prediction interval indicates that outcomes in future comparable cohorts may vary substantially. For late grade ≥3 GI toxicity, both strict-definition sensitivity analysis and a sparse-data alternative model yielded directionally similar estimates, suggesting that the clinically relevant toxicity signal was not solely driven by denominator choice or model specification. Taken together, these analyses support a signal of activity and a signal of clinically relevant late toxicity, but not a stable estimate that can yet be generalized across all non-operative rectal cancer settings.

The registered prospective landscape provides contextual support that this treatment concept remains under active evaluation, but it should be interpreted as background context rather than as additional outcome evidence. The ongoing studies summarized in Supplementary Table S5 underscore the need for better prospective standardization of response assessment, toxicity reporting, and durability endpoints. They also suggest that future progress will depend not only on larger cohorts but on clearer endpoint definitions and better alignment between clinical population, treatment intent, and outcome reporting.

This study has several limitations. First, the evidence base was small and consisted mainly of retrospective or single-center series, with only limited prospective comparative data. Second, clinical heterogeneity was substantial across patient populations, treatment pathways, and reporting practices. Third, outcome definitions were not uniform, particularly for local regrowth/local failure and for the denominators used in response and toxicity analyses. Fourth, follow-up was variable and, in several studies, insufficient for robust assessment of sustained local control or late morbidity. Finally, the pooled analyses were restricted to proportions rather than comparative treatment effects, and the present study cannot establish superiority over other organ-preservation strategies or over standard surgical management. These issues are relevant not only to the interpretation of this specific strategy but also to the broader design of prospective organ-preservation studies in rectal cancer, where response, durability, toxicity, and salvageability must be reported in a more integrated and clinically comparable way.

The present review also has several strengths. First, it focuses on a narrowly defined treatment strategy—definitive-intent EBRT, with or without chemotherapy, followed by HDR endorectal/endoluminal brachytherapy boost—rather than combining this approach with contact X-ray brachytherapy, planned preoperative brachytherapy, or mixed watch-and-wait cohorts. Second, it explicitly separates early response, late severe GI toxicity, and regrowth/local failure, thereby avoiding a misleading pooled estimate for outcomes with incompatible definitions and denominators. Third, denominator types were extracted and reported to improve transparency in the interpretation of response, toxicity, and local-failure outcomes. Finally, the review identifies practical methodological gaps that future prospective trials should address, including standardized endpoint definitions, durable local-control reporting, toxicity assessment, and population stratification.

The findings also highlight priorities for future studies: standardized denominator definitions, consistent reporting of late toxicity, formal time-to-event analysis of local regrowth and organ preservation, and prospective evaluation in clearly defined and prospectively stratified clinical populations.

## 5. Conclusions

Current evidence suggests that EBRT plus HDR endorectal/endoluminal brachytherapy boost may represent a selective organ-preservation strategy for carefully chosen patients with rectal adenocarcinoma, particularly where surgery is not feasible or not desired. Its broader clinical use remains limited not only by the small size of the evidence base, but also by fragmented endpoint definitions, inconsistent denominator reporting, and insufficiently standardized durable local-control outcomes. These findings support cautious interpretation of the current evidence and highlight priorities for future prospective studies in rectal cancer management.

## Figures and Tables

**Table 1:** Study characteristics and baseline context of included cohorts.

Study	Design	Setting	*N*	Clinical Intent	Population (as Reported)	Eligibility/Stage (as Reported)	Median Follow-Up	Unit
HERBERT cohort (Rijkmans 2017) [12]	Phase I prospective dose-escalation	Single-center	38	Inoperable/frail/refusal	Elderly/medically inoperable rectal cancer; some refusal	Rectal adenocarcinoma	30.0	months
Chiang 2020 [23]	Retrospective cohort/short communication	Two centers	18	Inoperable/frail	Medically unfit for surgery	cT2-4N0-2	18.2	months
IGAEBT registry (Garant 2019) [11]	Registry cohort	Multi-center/registry	94	Inoperable/refusal	Ineligible for surgery or refusing surgery	Rectal cancer	22.8	months
MORPHEUS (IGAEBT arm, 2022) [21]	Randomized phase II-III interim analysis	Multi-center	20	Operable, organ preservation	Operable rectal cancer; randomized HDR arm	cT2-3ab N0 M0	15.6	months (toxicity median 28.8 months; efficacy median 15.6 months)
Frankfurt cohort (Fleischmann 2022) [22]	Case series/first clinical experience	Single-center	6	Mixed inoperable/refusal	Elderly/frail; unfit or refused surgery	Rectal cancer	9.7	months
NOM-3 (Żółciak-Siwińska 2025) [10]	Prospective cohort (published)	Single-center	32	Operable, intentional NOM	Fit for radical surgery; intentional NOM	Tumors ≤5 cm in length and ≤50% of the circumference	22.0	months

Abbreviations: EBRT, external beam radiotherapy; HDR, high-dose-rate; HDR-BT, high-dose-rate brachytherapy; IGAEBT, image-guided adaptive endorectal brachytherapy; fx, fractions; FOLFOX4, folinic acid, fluorouracil, and oxaliplatin; NR, not reported; NA, not applicable.

**Table 2:** EBRT/chemotherapy and HDR endorectal/endoluminal boost parameters.

Study	EBRT Regimen	Chemotherapy	HDR Endorectal/Endoluminal Boost	Planning/Imaging	Prescription (Depth/Surface)
HERBERT cohort (Rijkmans 2017) [12]	39 Gy/13 fx	No concurrent chemo reported	HDREBT 5–8 Gy × 3 weekly; recommended 7 Gy/fx	Endorectal applicator; response assessed by endoscopy/MRI.	Weekly boost; prescription depth not consistently reported.
Chiang 2020 [23]	Mixed: 25/5, 39/13, 40/16, 50.4/28	None reported	HDREBT 10 Gy × 1–3	Image-guided HDREBT boost after EBRT.	10 Gy × 1–3; depth/surface not consistently reported.
IGAEBT registry (Garant 2019) [11]	40 Gy/16 fx	Radiotherapy alone	Adaptive IGAEBT 30 Gy/3 fx	Adaptive endorectal BT targeting residual tumor.	Adaptive boost to residual tumor; depth/surface not consistently reported.
MORPHEUS (IGAEBT arm, 2022) [21]	45 Gy/25 fx	5-FU/capecitabine	Adaptive IGAEBT 30 Gy/3 fx	Adaptive endorectal BT arm within phase II–III randomized trial.	30 Gy/3 fractions; depth/surface not consistently reported.
Frankfurt cohort (Fleischmann 2022) [22]	30 Gy/10 fx or 39 Gy/13 fx	None reported	HDR-BT 6 Gy × 2–3	Image-guided endorectal HDR-BT; endoscopy/MRI response evaluation.	Prescription at 5 mm (T1) or 10 mm (≥T2) from applicator surface (as reported); total 12–18 Gy in 2–3 fx.
NOM-3 (Żółciak-Siwińska 2025) [10]	Short-course RT 25 Gy/5 fx	3 cycles FOLFOX4	Adaptive HDR-BT planned 20 Gy/2 fx (10 Gy/fx); some got 1 fx	Endorectal HDR-BT boost within a planned non-operative strategy.	Planned 20 Gy/2 fractions (10 Gy/fx); some received 1 fraction (depth/surface per protocol).

Abbreviations: EBRT, external beam radiotherapy; HDR, high-dose-rate; HDR-BT, high-dose-rate brachytherapy; HDREBT, high-dose-rate endorectal brachytherapy; IGAEBT, image-guided adaptive endorectal brachytherapy; fx, fractions; 5-FU, fluorouracil; FOLFOX4, folinic acid, fluorouracil, and oxaliplatin; MRI, magnetic resonance imaging; NR, not reported; NA, not applicable.

**Table 3:** Key outcomes and denominator types in the main quantitative evidence base.

Study	cCR (*n*/*N*)	cCR Denominator Type	Late ≥ G3 GI (*n*/*N*)	Late ≥ G3 GI Denominator Type	Regrowth/Local Failure (*n*/*N*)	Median Follow-Up	Notes
HERBERT cohort (Rijkmans 2017) [12]	20/33	response-evaluable	10/32	toxicity-evaluable	12/33	30.0 months	Includes 6 recurrences after CR and 6 progressions after PR; 9 grade 3 and 1 grade 4 late toxicities.
IGAEBT registry (Garant 2019) [11]	81/94	treated (ITT)	12/94	treated (as reported)	11/81	22.8 months	Grade 3 late rectal bleeding reported in 12/94.
Chiang 2020 [23]	NR	NR	3/16	responders only	NR	18.2 months	Late toxicity was reported only in responders with follow-up.
MORPHEUS (IGAEBT arm, 2022) [21]	18/20	treated (ITT)	2/20	treated (ITT)	1/20	15.6 months (toxicity median 28.8 months; efficacy median 15.6 months)	Randomized HDR-IGAEBT arm.
Frankfurt cohort (Fleischmann 2022) [22]	4/6	treated (ITT)	0/6	treated (ITT)	0/6	9.7 months (42 weeks)	First clinical experience cohort; no grade ≥3 GI toxicity or local failure reported.
NOM-3 2025 [10]	11/32	treated (ITT)	NR	NR	8/25	22.0 months	Local failure count was back-calculated from the reported 34% among 25 evaluable patients.

Abbreviations: cCR, clinical complete response; GI, gastrointestinal; CR, complete response; PR, partial response; ITT, intention-to-treat; NR, not reported; NA, not applicable. Denominator definitions: treated (ITT), all treated patients; response-evaluable, patients assessable for tumor response; toxicity-evaluable, patients assessable for toxicity; responders only, patients with reported response subsets only.

## Data Availability

The data supporting the findings of this study are available from the published articles included in this systematic review and meta-analysis. Additional extracted data are provided in the article and Supplementary Material.

## Supplementary Materials

The following supporting information can be downloaded at: https://www.mdpi.com/article/10.3390/cancers18091494/s1, Figure S1: Risk of bias summary for non-randomized cohorts assessed using the JBI Case Series checklist; Figure S2: Leave-one-out analysis for pooled clinical complete response; Figure S3: Leave-one-out analysis for pooled late grade ≥3 gastrointestinal toxicity; Table S1: Predefined sensitivity sets and rationale; Table S2: Main and sensitivity pooled estimates for clinical complete response; Table S3: Main and strict-definition sensitivity pooled estimates for late grade ≥3 gastrointestinal toxicity; Table S4: Main and alternative sparse-data model estimates for late grade ≥3 gastrointestinal toxicity; Table S5: Registered prospective studies related to endorectal/endoluminal HDR brachytherapy in rectal cancer (ClinicalTrials.gov query date, 6 March 2026).
