# Supplementary material for: HDR Endorectal/Endoluminal Brachytherapy Boost in Rectal Organ Preservation: A Systematic Review and Meta-Analysis
Source: Cancers (Basel). 2026 May 6;18(9):1494. doi: 10.3390/cancers18091494 (PMC13163075; doi:10.3390/cancers18091494)
Supplement: Supplementary file 1 [file cancers-18-01494-s001.zip › cancers-4284886-supplementary/cancers-4284886-suppl-conversion/cancers-4284886-suppl-conversion.pdf]

cancers

MDPI

Supplementary Material

HDR Endorectal/Endoluminal Brachytherapy Boost in Rectal Organ Preservation: A Systematic Review and Meta-Analysis

Yuanjie Cao, Chen Li, Baozhong Zhang * and Jie Chen *

Supplementary Methods

S1. Database search strategies

Database searches were finalized on 6 March 2026. Searches were performed in PubMed, Embase, and the Cochrane Central Register of Controlled Trials (CENTRAL). In addition, ClinicalTrials.gov was queried on the same date to identify registered prospective studies relevant to the review question. The review protocol was registered in PROSPERO (CRD420261339587). The search strategy combined terms for rectal cancer, rectal adenocarcinoma, external beam radiotherapy/chemoradiotherapy, high-dose-rate brachytherapy, and endorectal/endoluminal brachytherapy, together with broader organ-preservation/non-operative management terminology when appropriate. No language restrictions were applied during the initial search.

S1.1 PubMed

((“Rectal Neoplasms”[Mesh] OR rectal cancer[tiab] OR rectal carcinoma[tiab] OR rectal adenocarcinoma[tiab]))

AND

((“Radiotherapy”[Mesh] OR “Chemoradiotherapy”[tiab] OR chemoradiation[tiab] OR chemoradiotherapy[tiab] OR radiotherapy[tiab] OR radiation therapy[tiab] OR external beam radiotherapy[tiab] OR EBRT[tiab] OR external beam radiation[tiab] OR CRT[tiab] OR CCRT[tiab]))

AND

((“Brachytherapy”[Mesh] OR brachytherapy[tiab] OR high-dose-rate brachytherapy[tiab] OR HDR brachytherapy[tiab] OR HDR-BT[tiab] OR endorectal brachytherapy[tiab] OR endoluminal brachytherapy[tiab] OR intraluminal brachytherapy[tiab] OR contact brachytherapy[tiab]))

AND

((endorectal[tiab] OR endoluminal[tiab] OR intraluminal[tiab] OR boost[tiab]))

NOT

((anal cancer[tiab] OR anal carcinoma[tiab]))

For reporting consistency and to focus on contemporary HDR endorectal/endoluminal brachytherapy practice, modern imaging-based rectal cancer assessment, and organ-preservation frameworks relevant to current multidisciplinary management, the final search was limited to publications from 1 January 2010 to 6 March 2026.

S1.2 Embase

(‘rectum cancer’/exp OR ‘rectal cancer’:ti,ab OR ‘rectal carcinoma’:ti,ab OR ‘rectal adenocarcinoma’:ti,ab)

AND

 https://doi.org/10.3390/cancers18091494

(‘radiotherapy’/exp OR ‘chemoradiotherapy’/exp OR chemoradiotherapy:ti,ab OR chemoradiation:ti,ab OR radiotherapy:ti,ab OR ‘radiation therapy’:ti,ab OR ‘external beam radiotherapy’:ti,ab OR EBRT:ti,ab OR CRT:ti,ab OR CCRT:ti,ab)

AND

(‘brachytherapy’/exp OR brachytherapy:ti,ab OR ‘high dose rate brachytherapy’:ti,ab OR ‘high-dose-rate brachytherapy’:ti,ab OR ‘HDR brachytherapy’:ti,ab OR HDR-BT:ti,ab OR ‘endorectal brachytherapy’:ti,ab OR ‘endoluminal brachytherapy’:ti,ab OR ‘intraluminal brachytherapy’:ti,ab OR ‘contact brachytherapy’:ti,ab)

AND

(endorectal:ti,ab OR endoluminal:ti,ab OR intraluminal:ti,ab OR boost:ti,ab)

NOT

(‘anal cancer’:ti,ab OR ‘anal carcinoma’:ti,ab)

AND

[2010-2026]/py

S1.3 Cochrane CENTRAL

(rectal cancer OR rectal carcinoma OR rectal adenocarcinoma)

AND

(radiotherapy OR chemoradiotherapy OR chemoradiation OR “external beam radiotherapy” OR EBRT OR CRT OR CCRT)

AND

(brachytherapy OR “high-dose-rate brachytherapy” OR “HDR brachytherapy” OR “endorectal brachytherapy” OR “endoluminal brachytherapy” OR “intraluminal brachytherapy”)

AND

(endorectal OR endoluminal OR intraluminal OR boost)

NOT

(anal cancer OR anal carcinoma)

The final search was restricted to studies published between 2010 and 2026 for consistency with the prespecified contemporary evidence window used in the main review.

S1.4 ClinicalTrials.gov

ClinicalTrials.gov was searched to identify relevant registered prospective studies and ongoing trials.

Basic search string:

(rectal cancer OR rectal adenocarcinoma) AND (brachytherapy OR endorectal brachytherapy OR endoluminal brachytherapy OR HDR brachytherapy)

Broader alternative search string:

(rectal cancer) AND (organ preservation OR non-operative management OR watch and wait OR brachytherapy OR endorectal OR endoluminal)

Suggested filters applied at the screening stage included interventional studies and relevant study status categories (recruiting, active not recruiting, and completed). ClinicalTrials.gov was queried on 6 March 2026.

S1.5 Notes on strategy development

The search strategy was designed to maximize sensitivity for studies evaluating definitive-intent external beam radiotherapy with HDR endorectal/endoluminal brachytherapy boost in rectal cancer, while final eligibility focused on histologically confirmed rectal adenocarcinoma managed without planned surgery. Broader terms related to or-

gan preservation and non-operative management were used mainly to support contextual trial identification, whereas final inclusion was determined through manual screening against prespecified eligibility criteria. Studies focused primarily on anal squamous cell carcinoma, anal canal cancer, non-adenocarcinoma anorectal malignancies, contact X-ray brachytherapy, planned surgery after neoadjuvant treatment, or non-extractable mixed populations were excluded during screening rather than through overly restrictive database syntax.

S2. Risk of bias assessment

Single-arm non-randomized cohorts/series were assessed using the Joanna Briggs Institute (JBI) Critical Appraisal Checklist for Case Series. The randomized trial arm was assessed using RoB 2. Concise supporting evidence from each full text is provided below.

S2.1 JBI Case Series: item-level judgements with supporting evidence

Legend: ✓yes; ? unclear; ✗ no.

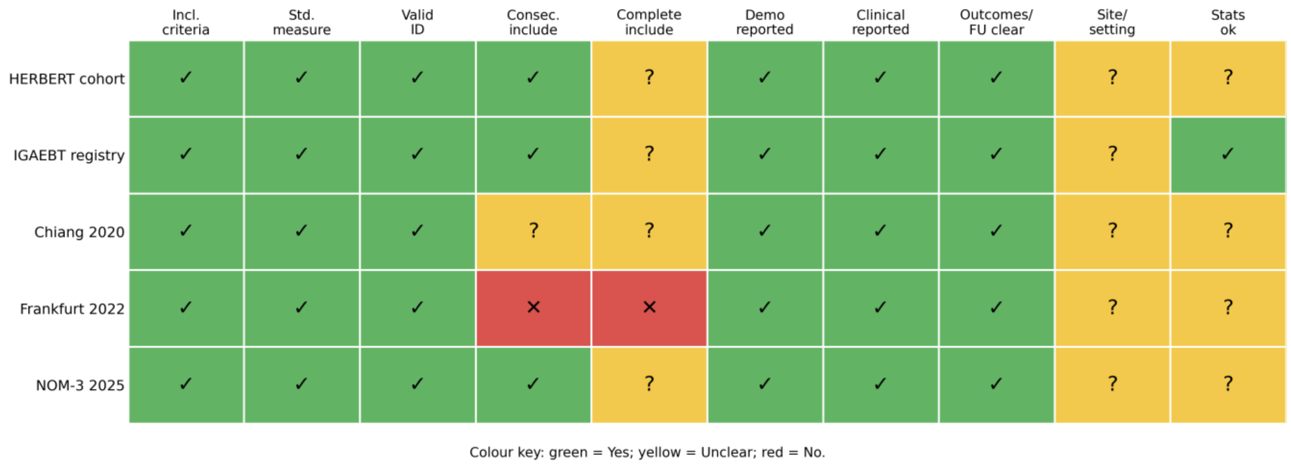

Figure S1. Risk of bias summary for non-randomized cohorts assessed using the JBI Case Series checklist. *Colour key: green = yes; yellow = unclear; red = no.*

S2.2 RoB 2: randomized trial arm (MORPHEUS) summary

The randomized trial arm (MORPHEUS) was assessed using RoB 2 and judged as having some concerns overall. Registry information and the interim report supported randomization, but allocation concealment and details of outcome measurement were insufficiently reported for a low-risk judgement. RoB 2 is therefore presented as a narrative summary because only a single trial arm was eligible for that assessment.

Overall RoB 2 judgement: some concerns.

Supplementary Robustness Analyses

This section provides prespecified robustness analyses for the pooled clinical complete response (cCR) and late grade ≥3 gastrointestinal (GI) toxicity endpoints, together with the registered prospective trial landscape referenced in the main manuscript. Each sensitivity analysis used endpoint-specific available study sets as prespecified in Supplementary Table S1 (cCR main set n=5; late grade ≥3 GI toxicity main set n = 5).

https://doi.org/10.3390/cancers18091494

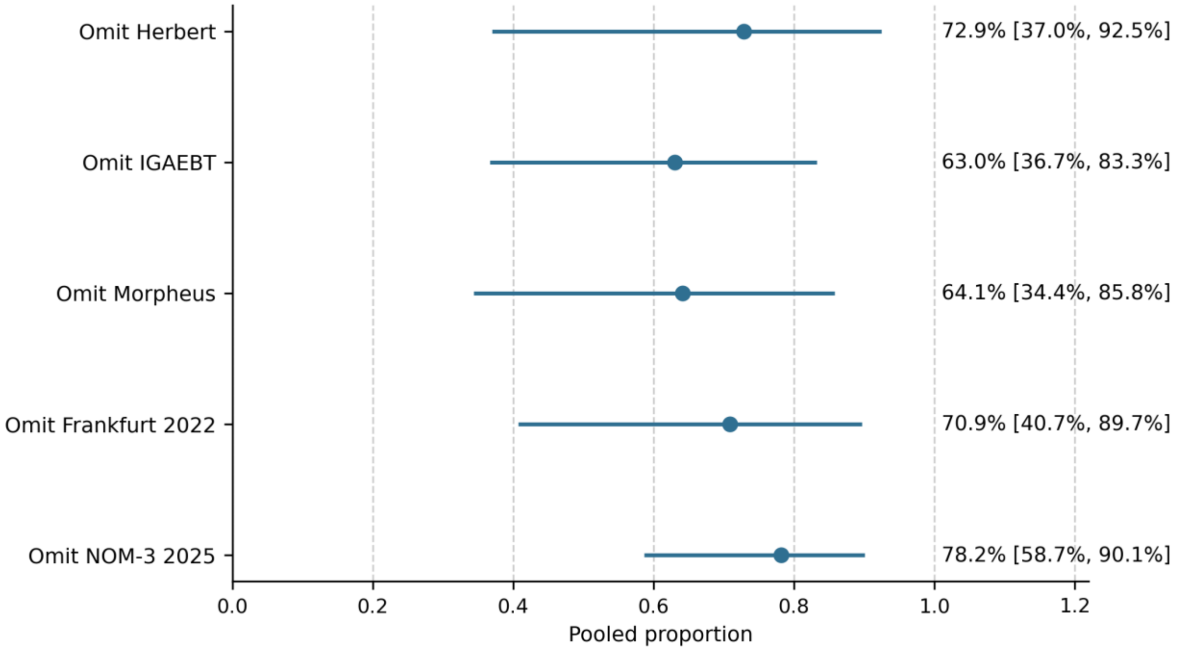

Figure S2. Leave-one-out analysis for pooled clinical complete response. *Sequential omission of individual studies showed that the pooled cCR signal remained directionally consistent, although the magnitude of the pooled estimate varied across iterations.*

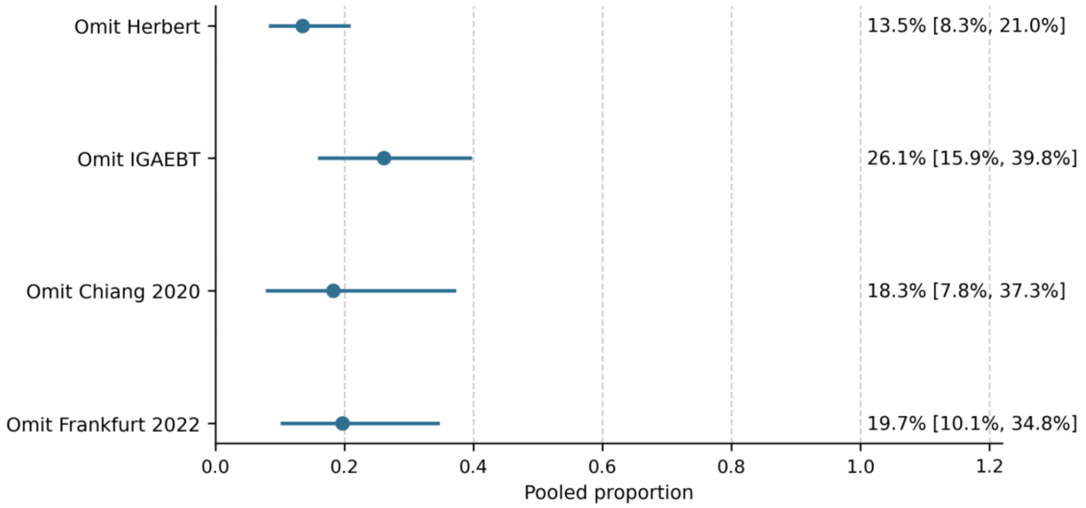

Figure S3. Leave-one-out analysis for pooled late grade ≥3 gastrointestinal toxicity. *Sequential omission analyses suggested that no single study fully accounted for the pooled late grade ≥3 GI toxicity estimate.*

Table S1. Predefined sensitivity sets and rationale.

Endpoint	Primary dataset	Sensitivity dataset	Rationale
cCR	HERBERT (Rijkmans 2017); Garant 2019; MORPHEUS IGAEBT 2022; Frankfurt 2022; NOM-3	HERBERT (Rijkmans 2017); Garant 2019; MORPHEUS IGAEBT 2022; NOM-3 2025 (n = 4)	Excludes the smallest early feasibility-style cohort to assess small-study influence.

https://doi.org/10.3390/cancers18091494

	2025 (n = 5)		
Late grade ≥3 GI toxicity	HERBERT (Rijkmans 2017); Garant 2019; Chiang 2020; MORPHEUS IGAEBT 2022; Frankfurt 2022 (n = 5)	HERBERT (Rijkmans 2017); Garant 2019; MORPHEUS IGAEBT 2022; Frankfurt 2022 (n = 4)	Excludes the study reporting toxicity with responder-only denominators to test denominator sensitivity.

Table S2. Main and sensitivity pooled estimates for clinical complete response.

Analysis	Studies included	Pooled cCR	95% CI	Additional information
Primary manuscript estimate (Figure 2A)	Main cCR synthesis set (n = 5)	69.2%	43.7–86.6%	$I^2 = 87.2\%$
Leave-one-out: omit HERBERT (Rijkmans 2017)	4 studies	72.9%	37.0–92.5%	$I^2 = 90.0\%$
Leave-one-out: omit Garant 2019	4 studies	63.0%	36.7–83.3%	$I^2 = 77.1\%$
Leave-one-out: omit MORPHEUS IGAEBT 2022	4 studies	64.1%	34.4–85.8%	$I^2 = 89.3\%$
Leave-one-out: omit Frankfurt 2022	4 studies	70.9%	40.7–89.7%	$I^2 = 90.5\%$
Leave-one-out: omit NOM-3 2025	4 studies	78.2%	58.7–90.1%	$I^2 = 72.8\%$
Best-evidence sensitivity set	Exclude Frankfurt 2022	70.9%	40.7–89.7%	$I^2 = 90.5\%$
Prediction interval	Main cCR synthesis set (n = 5)	-	–	95% prediction interval approximately 16.9–96.1%

Table S3. Main and strict-definition sensitivity pooled estimates for late grade ≥3 gastrointestinal toxicity.

Analysis	Studies included	Pooled toxicity	95% CI	Additional information
Primary manuscript estimate (Figure 2B)	Main toxicity synthesis set (n = 5)	18.1%	10.9–28.6%	$I^2 = 40.0\%$
Leave-one-out: omit HERBERT (Rijkmans 2017)	4 studies	13.0%	8.3–19.8%	$I^2 = 0.0\%$
Leave-one-out: omit Garant 2019	4 studies	20.5%	11.0–35.1%	$I^2 = 54.5\%$
Leave-one-out: omit	4 studies	16.6%	8.2–30.5%	$I^2 = 50.4\%$

Chiang 2020				
Leave-one-out: omit MORPHEUS IGAEBT 2022	4 studies	18.6%	10.1–31.8%	$I^2 = 51.0\%$
Leave-one-out: omit Frankfurt 2022	4 studies	17.8%	10.0–29.8%	$I^2 = 37.0\%$
Strict-definition sensitivity set	Exclude Chiang 2020	17.3%	8.9–31.0%	$I^2 = 54.5\%$

Table S4. Main and alternative sparse-data model estimates for late grade ≥3 gastrointestinal toxicity.

Model	Studies included	Pooled toxicity	95% model-based interval	Notes
Primary manuscript estimate (Figure 2B)	Main toxicity synthesis set (n = 5)	18.1%	10.9–28.6%	Random-effects proportion meta-analysis is reported in the main manuscript.
Alternative sparse-data model	Main toxicity synthesis set (n = 5)	17.3%	10.0–26.0%	Directionally similar result using a sparse-data sensitivity framework.

Table S5. Registered prospective studies related to endorectal/endoluminal HDR brachytherapy in rectal cancer (ClinicalTrials.gov query date, 6 March 2026).

Trial ID	Study	Status	Design	Clinical setting	HDR component	Main relevance
NCT03051464	MORPHEUS	Recruiting	Randomized organ-preservation trial	cT2-3 rectal cancer after CRT	HDR boost vs EBRT boost	Directly matches the review question
NCT04927897	DETECT	Recruiting	Prospective observational study	Candidates for endoluminal boost	Boost target-volume definition	Technical standardization
NCT06087718	Maastro feasibility	Active, not recruiting	Pilot interventional study	Patients receiving an endoluminal boost	Maastro HDR/contact platform	Applicator feasibility
NCT07402486	Maastro phase II	Not yet recruiting	Single-arm phase II study	Selected organ-preservation candidates	Maastro-based HDR brachytherapy	Prospective modern HDR evaluation

Abbreviations: CRT, chemoradiotherapy; EBRT, external beam radiotherapy; HDR, high-dose-rate. Trial information was summarized from ClinicalTrials.gov using a registry extraction per-

formed on 6 March 2026. These studies were not included in the pooled analyses and are presented to contextualize ongoing prospective development in this field.

Disclaimer/Publisher's Note: The statements, opinions and data contained in all publications are solely those of the individual author(s) and contributor(s) and not of MDPI and/or the editor(s). MDPI and/or the editor(s) disclaim responsibility for any injury to people or property resulting from any ideas, methods, instructions or products referred to in the content.

https://doi.org/10.3390/cancers18091494
