# Supplementary material for: HDR Endorectal/Endoluminal Brachytherapy Boost in Rectal Organ Preservation: A Systematic Review and Meta-Analysis
Source: Cancers (Basel). 2026 May 6;18(9):1494. doi: 10.3390/cancers18091494 (PMC13163075; doi:10.3390/cancers18091494)
Supplement: Supplementary file 1 [file cancers-18-01494-s001.zip › cancers-4284886-supplementary/cancers-4284886-suppl-conversion/PRISMA_2020_checklist_completed-2026.05.06.pdf]

PRISMA 2020 Checklist

Section and Topic	Item #	Checklist item	Location where item is reported
**TITLE**			
Title	1	Identify the report as a systematic review.	Title page, p. 1: title identifies the report as a systematic review and meta-analysis.
**ABSTRACT**			
Abstract	2	See the PRISMA 2020 for Abstracts checklist.	Abstract, pp. 2–3: structured abstract reports background/purpose, methods, results, and conclusion.
**INTRODUCTION**			
Rationale	3	Describe the rationale for the review in the context of existing knowledge.	Introduction, pp. 3–4: rationale and clinical context of organ preservation and HDR endorectal/endoluminal brachytherapy boost.
Objectives	4	Provide an explicit statement of the objective(s) or question(s) the review addresses.	Introduction, p. 4: final paragraph states the objectives of synthesizing cCR, severe late GI toxicity, clinical positioning, and methodological barriers.
**METHODS**			
Eligibility criteria	5	Specify the inclusion and exclusion criteria for the review and how studies were grouped for the syntheses.	Methods, Section 2.2, p. 4: eligibility criteria, inclusion/exclusion criteria, and grouping by non-operative intent.
Information sources	6	Specify all databases, registers, websites, organisations, reference lists and other sources searched or consulted to identify studies. Specify the date when each source was last searched or consulted.	Methods, Section 2.3, pp. 5–6; Supplementary Methods: PubMed, Embase, CENTRAL, ClinicalTrials.gov, reference lists, and search date are reported.
Search strategy	7	Present the full search strategies for all databases, registers and websites, including any filters and limits used.	Methods, Section 2.3, pp. 5–6; Supplementary Methods/Supplementary Material: full database search strategies, filters, and limits are reported.
Selection process	8	Specify the methods used to decide whether a study met the inclusion criteria of the review, including how many reviewers screened each record and each report retrieved, whether they worked independently, and if applicable, details of automation tools used in the process.	Methods, Section 2.3, p. 5: two reviewers independently screened titles/abstracts and full texts; disagreements were resolved by discussion/adjudication by a third reviewer.
Data collection process	9	Specify the methods used to collect data from reports, including how many reviewers collected data from each report, whether they worked independently, any processes for obtaining or confirming data from study investigators, and if applicable, details of automation tools used in the process.	Methods, Section 2.3, p. 5 and Section 2.4, p. 5: standardized extraction form, cross-checking for accuracy, and extracted variables are described.
Data items	10a	List and define all outcomes for which data were sought. Specify whether all results that were compatible with each outcome domain in each study were sought (e.g. for all measures, time points, analyses), and if not, the methods used to decide which results to collect.	Methods, Section 2.4, p. 5: primary endpoints were cCR and late grade ≥3 GI toxicity; regrowth/local failure, follow-up, and denominator type were extracted descriptively.
	10b	List and define all other variables for which data were sought (e.g. participant and intervention characteristics, funding sources). Describe any assumptions made about any missing or unclear information.	Methods, Section 2.4, p. 5; Tables 1–3, pp. 7–8: study design, cohort context, EBRT/chemotherapy details, HDR boost parameters, follow-up, denominator type, and outcome data are reported.
Study risk of bias assessment	11	Specify the methods used to assess risk of bias in the included studies, including details of the tool(s) used, how many reviewers assessed each study and whether they worked independently, and if applicable, details of automation tools used in the process.	Methods, Section 2.5, p. 5: JBI checklist and RoB 2 are specified; two reviewers independently assessed risk of bias, with adjudication as needed. Supplementary Figure S1 provides the visual summary.
Effect measures	12	Specify for each outcome the effect measure(s) (e.g. risk ratio, mean difference) used in the synthesis or presentation of results.	Methods, Section 2.6, pp. 5–6: proportions with 95% confidence intervals were used for pooled and descriptive outcome presentation.
Synthesis methods	13a	Describe the processes used to decide which studies were eligible for each synthesis (e.g. tabulating the study intervention characteristics and comparing against the planned groups for each synthesis (item #5)).	Methods, Sections 2.2–2.6, pp. 4–6; Results, Section 3.3, pp. 8–9: endpoint-specific available study sets and denominator structures are described.
	13b	Describe any methods required to prepare the data for presentation or synthesis, such as handling of missing summary statistics, or data conversions.	Methods, Sections 2.4 and 2.6, pp. 5–6; Results, Sections 3.3–3.4, pp. 8–12: extraction of numerator/denominator data, back-calculation where necessary, and descriptive handling of non-comparable regrowth/local failure outcomes are reported.
	13c	Describe any methods used to tabulate or visually display results of individual studies and syntheses.	Methods, Section 2.6, pp. 5–6; Results, Sections 3.3–3.4, pp. 8–12; Figures 2–3 and Tables 1–3: tabular and graphical display methods are described and implemented.
	13d	Describe any methods used to synthesize results and provide a rationale for the choice(s). If meta-analysis was performed, describe the model(s), method(s) to identify the presence and	Methods, Section 2.6, pp. 5–6: random-effects logit models, DerSimonian-Laird τ^2 estimation, I^2, and R version 4.5.1 are reported.

Section and Topic	Item #	Checklist item	Location where item is reported
		extent of statistical heterogeneity, and software package(s) used.	
	13e	Describe any methods used to explore possible causes of heterogeneity among study results (e.g. subgroup analysis, meta-regression).	Methods, Section 2.6, pp. 5–6; Discussion, pp. 15–18: heterogeneity was quantified with I^2 and explored descriptively because of limited study numbers and clinical/reporting heterogeneity.
	13f	Describe any sensitivity analyses conducted to assess robustness of the synthesized results.	Methods, Section 2.6, p. 6; Results, Section 3.6, p. 14; Supplementary Figures S2–S3 and Tables S2–S4: leave-one-out, strict-definition, prediction interval, and sparse-data sensitivity analyses are reported.
Reporting bias assessment	14	Describe any methods used to assess risk of bias due to missing results in a synthesis (arising from reporting biases).	Not formally assessed with funnel plots or statistical tests because of the small number of included studies per synthesis; this limitation is addressed in the Discussion, pp. 17–18.
Certainty assessment	15	Describe any methods used to assess certainty (or confidence) in the body of evidence for an outcome.	No formal GRADE/certainty assessment was performed; risk of bias and evidence limitations are reported in Methods 2.5, Supplementary Figure S1, and Discussion, pp. 17–18.
**RESULTS**			
Study selection	16a	Describe the results of the search and selection process, from the number of records identified in the search to the number of studies included in the review, ideally using a flow diagram.	Results, Section 3.1, p. 6 and Figure 1, p. 6: study selection numbers and PRISMA flow diagram are reported.
	16b	Cite studies that might appear to meet the inclusion criteria, but which were excluded, and explain why they were excluded.	Results, Section 3.1, p. 6 and Figure 1, p. 6: full-text exclusions and reasons are reported; additional context is provided in Supplementary Material.
Study characteristics	17	Cite each included study and present its characteristics.	Results, Section 3.2, p. 7; Tables 1–2, pp. 7–8: each included study is cited and study/treatment characteristics are presented.
Risk of bias in studies	18	Present assessments of risk of bias for each included study.	Methods, Section 2.5, p. 5; Supplementary Figure S1 and Supplementary Material: study-level risk-of-bias assessments are presented.
Results of individual studies	19	For all outcomes, present, for each study: (a) summary statistics for each group (where appropriate) and (b) an effect estimate and its precision (e.g. confidence/credible interval), ideally using structured tables or plots.	Results, Sections 3.3–3.4, pp. 8–12; Table 3; Figures 2–3: study-level numerators/denominators, proportions, and confidence intervals are presented.
Results of syntheses	20a	For each synthesis, briefly summarise the characteristics and risk of bias among contributing studies.	Results, Sections 3.2–3.3, pp. 7–9; Tables 1–3: characteristics, denominator types, and risk-of-bias context for studies contributing to each synthesis are summarized.
	20b	Present results of all statistical syntheses conducted. If meta-analysis was done, present for each the summary estimate and its precision (e.g. confidence/credible interval) and measures of statistical heterogeneity. If comparing groups, describe the direction of the effect.	Results, Section 3.3, pp. 8–11; Figure 2: pooled cCR and late grade ≥3 GI toxicity estimates with 95% confidence intervals and I^2 are reported.
	20c	Present results of all investigations of possible causes of heterogeneity among study results.	Results, Section 3.5, p. 13; Discussion, pp. 15–18: heterogeneity and its clinical/reporting sources are interpreted descriptively.
	20d	Present results of all sensitivity analyses conducted to assess the robustness of the synthesized results.	Results, Section 3.6, p. 14; Supplementary Figures S2–S3 and Tables S2–S4: robustness and sensitivity analyses are reported.
Reporting biases	21	Present assessments of risk of bias due to missing results (arising from reporting biases) for each synthesis assessed.	Not formally assessed because of the small number of studies; possible reporting heterogeneity and missing/inconsistent outcomes are discussed in Results 3.4–3.5 and Discussion, pp. 15–18.
Certainty of evidence	22	Present assessments of certainty (or confidence) in the body of evidence for each outcome assessed.	No formal certainty assessment was undertaken; the limitations of the evidence base and implications for confidence in the findings are discussed in Discussion, pp. 17–18.
**DISCUSSION**			
Discussion	23a	Provide a general interpretation of the results in the context of other evidence.	Discussion, pp. 14–19: interpretation in context of OPRA/OPERA and the broader organ-preservation literature is provided.
	23b	Discuss any limitations of the evidence included in the review.	Discussion, pp. 17–18: limitations of the included evidence, including small cohorts, heterogeneity, denominators, and follow-up, are discussed.
	23c	Discuss any limitations of the review processes used.	Discussion, pp. 17–18: limitations of the review process and synthesis constraints are discussed.
	23d	Discuss implications of the results for practice, policy, and future research.	Discussion, pp. 18–19 and Conclusion, p. 19: implications for selective clinical use and future prospective studies are discussed.

PRISMA

PRISMA 2020 Checklist

Section and Topic	Item #	Checklist item	Location where item is reported
**OTHER INFORMATION**			
Registration and protocol	24a	Provide registration information for the review, including register name and registration number, or state that the review was not registered.	Methods, Section 2.1, p. 4: PROSPERO registration is reported as CRD420261339587.
	24b	Indicate where the review protocol can be accessed, or state that a protocol was not prepared.	Methods, Section 2.1, p. 4: registration in PROSPERO is reported; protocol access is through PROSPERO record CRD420261339587.
	24c	Describe and explain any amendments to information provided at registration or in the protocol.	No protocol amendments requiring separate reporting were made; methods are reported according to the final registered protocol and manuscript methods.
Support	25	Describe sources of financial or non-financial support for the review, and the role of the funders or sponsors in the review.	Funding statement, p. 19: Tianjin Medical University Cancer Institute & Hospital "358 Project" grant numbers 358-2022-11 and 358-2023-3; APC funding is reported.
Competing interests	26	Declare any competing interests of review authors.	Conflicts of Interest statement, p. 20: the authors declare no conflicts of interest.
Availability of data, code and other materials	27	Report which of the following are publicly available and where they can be found: template data collection forms; data extracted from included studies; data used for all analyses; analytic code; any other materials used in the review.	Data Availability Statement, p. 20; Supplementary Material: data are available from the included published articles, the article, and Supplementary Material.

From: Page MJ, McKenzie JE, Bossuyt PM, Boutron I, Hoffmann TC, Mulrow CD, et al. The PRISMA 2020 statement: an updated guideline for reporting systematic reviews. BMJ 2021;372:n71. doi: 10.1136/bmj.n71. This work is licensed under CC BY 4.0. To view a copy of this license, visit https://creativecommons.org/licenses/by/4.0/
